# Unfolding pathway and intermolecular interactions of the cytochrome subunit in the bacterial photosynthetic reaction center

**DOI:** 10.1016/j.bbabio.2020.148204

**Published:** 2020-08-01

**Authors:** Leanne C. Miller, Longsheng Zhao, Daniel P. Canniffe, David Martin, Lu-Ning Liu

**Affiliations:** a Institute of Integrative Biology, University of Liverpool, Liverpool L69 7ZB, United Kingdom; b Department of Physics, University of Liverpool, Liverpool L69 7ZE, United Kingdom; c State Key Laboratory of Microbial Technology, Marine Biotechnology Research Center, Shandong University, Qingdao 266237, China; d Laboratory for Marine Biology and Biotechnology, Pilot National Laboratory for Marine Science and Technology, Qingdao 266237, China; e College of Marine Life Sciences, and Frontiers Science Center for Deep Ocean Multispheres and Earth System, Ocean University of China, Qingdao 266003, China

**Keywords:** Atomic force microscopy, Cytochrome, Photosynthetic membrane, Protein unfolding, Reaction center, Single-molecule force spectroscopy

## Abstract

Precise folding of photosynthetic proteins and organization of multicomponent assemblies to form functional entities are fundamental to efficient photosynthetic electron transfer. The bacteriochlorophyll *b*-producing purple bacterium *Blastochloris viridis* possesses a simplified photosynthetic apparatus. The light-harvesting (LH) antenna complex surrounds the photosynthetic reaction center (RC) to form the RC-LH1 complex. A non-membranous tetraheme cytochrome (4Hcyt) subunit is anchored at the periplasmic surface of the RC, functioning as the electron donor to transfer electrons from mobile electron carriers to the RC. Here, we use atomic force microscopy (AFM) and single-molecule force spectroscopy (SMFS) to probe the long-range organization of the photosynthetic apparatus from *Blc. viridis* and the unfolding pathway of the 4Hcyt subunit in its native supramolecular assembly with its functional partners. AFM images reveal that the RC-LH1 complexes are densely organized in the photosynthetic membranes, with restricted lateral protein diffusion. Unfolding of the 4Hcyt subunit represents a multi-step process and the unfolding forces of the 4Hcyt α-helices are approximately 121 picoNewtons. Pulling of 4Hcyt could also result in the unfolding of the RC L subunit that binds with the N-terminus of 4Hcyt, suggesting strong interactions between RC subunits. This study provides new insights into the protein folding and interactions of photosynthetic multicomponent complexes, which are essential for their structural and functional integrity to conduct photosynthetic electron flow.

## Highlights

• AFM and single-molecule force spectroscopy reveal the membrane organization and unfolding process of *Blastochloris viridis* RC
• RC-light-harvesting 1 complexes are densely organized in photosynthetic membranes, with restricted lateral diffusion
• Unfolding of the non-membranous cytochrome (4Hcyt) subunit represents a multi-step process;
• The average unfolding forces of the 4Hcyt α-helices are ~121 pN;
• Pulling of 4Hcyt from the RC suggests strong interactions (> 150 pN) between 4Hcyt and L subunits in the RC structure.

## 1 Introduction

Photosynthesis is one of the most important biological processes, providing the energy for almost all life on Earth. The photosynthetic light reactions occur in the specialized biological membranes, termed the photosynthetic membranes, which accommodate a set of pigment–protein photosynthetic complexes. Light is absorbed by the pigments in the antenna complexes, and the energy is transferred efficiently to the reaction center (RC), where primary charge separation takes place and electron transfer is initiated. Defined protein organization and interaction in photosynthetic membranes are paramount to high-efficiency light-harvesting and energy conversion.

**Fig. 1:**
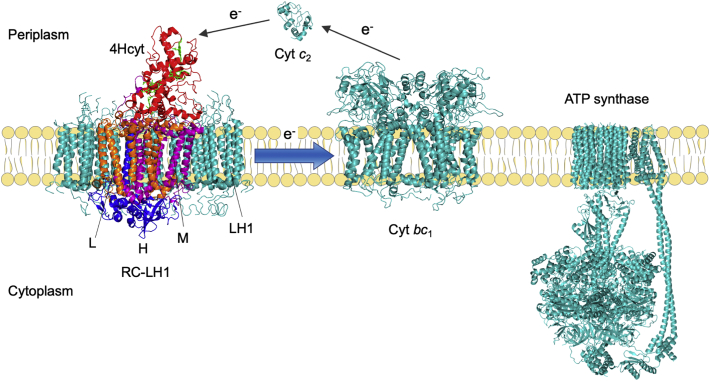
Schematic model of the photosynthetic apparatus of *Blc. viridis*. The electron transport chain of *Blc. viridis* comprises the RC-LH1 complex (PDB ID: 6ET5, *Blc. viridis*), Cyt *bc*_1_ (PDB ID: 1ZRT, *Rhodobacter capsulatus*), and ATP synthase (PDB ID: 6N30, *Bacillus* sp. strain PS3). The 4Hcyt subunit (red) at the periplasmic side of the photosynthetic RC contains four heme groups (green). It interacts with the transmembrane subunits L (orange) and M (purple). The H subunit is at the cytoplasmic side of the RC (blue). LH1 complexes (teal) associate with the RC, forming the RC-LH1 core complex. The Cyt *c*_2_ functions as the soluble electron carriers and transfers electrons from the Cyt *bc*_1_ complex to the RC.

In purple photosynthetic bacteria, the light-harvesting system is predominantly made up of two pigment–protein antennas: the peripheral light-harvesting complexes LH2 and the core light-harvesting complexes LH1. LH2 funnels the excitation energy to an LH1 complex that encircles an RC to form the RC–LH1 core complexes. *Blastochloris* (*Blc*.) *viridis* is an unusual, bacteriochlorophyll (BChl) *b*-producing purple bacterium, possessing a simplified photosynthetic apparatus that lacks LH2 [1]. The RC of *Blc. viridis* was the first membrane protein to be structurally characterized [2]. It is formed by four subunits (Fig. 1): three core subunits L (PufL), M (PufM), H (PuhA) that are bound to the cytoplasmic face of the L and M subunits, as well as a non-membranous tetraheme cytochrome subunit (4Hcyt, PufC) with its N-terminal cysteine covalently linked to a diglyceride at the periplasmic surface of the photosynthetic membrane; the 4Hcyt subunit is non-covalently linked to the L and M subunits [[3], [4], [5]]. In addition, the RC consists of four BChl *b* molecules, two bacteriopheophytin *b* molecules, one nonheme iron, two quinones, and one carotenoid [6]. The *Blc. viridis* LH1 complex is composed of α-, β- and γ-polypeptides. A recent cryo-electron microscopy structure of the *Blc. viridis* RC-LH1 complex revealed that the LH1 ring consists of 16 heterotrimers of α–β–γ-polypeptides and one α–β-heterodimer; the ‘missing’ 17th γ-polypeptide creates a gap in the LH1 ring, potentially allowing for quinone diffusion [7].

The 4Hcyt subunit contains four covalently bound heme groups and is attached to the periplasmic face of the L and M subunits, transferring electrons from the soluble electron carrier cytochrome (Cyt) *c*_2_ molecule to the RC “special pair” of BChls (Fig. 1). The conformation of the 4Hcyt subunit is designed for rapid electron flow between the vertically stacked heme groups and the binding of Cyt *c*_*2*_ to the RC is speculated to be the limiting step of electron transfer [8]. Recent structural analysis has provided information about the Cyt-RC association in several species [7,[9], [10], [11]]. However, the 4Hcyt subunit is not a crucial RC component in all purple photosynthetic bacteria. Some species, such as *Rhodobacter* (*Rba.*) *sphaeroides* [12], *Rhodospirillum rubrum* [13], and *Rhodopseudomonas palustris* [14], do not contain a 4Hcyt subunit and rely on Cyt *c*_2_ or other electron carriers (such as Cyt *c*_y_) for electron donation. Despite many structural studies at close to atomic resolution of bacterial RCs, how the 4Hcyt subunit folds to position its heme cofactors and interact with the RC subunits in the native complex remains enigmatic.

In this work, we apply atomic force microscopy (AFM) imaging and AFM-based single-molecule force spectroscopy (SMFS) to elucidate the native organization of the photosynthetic apparatus from *Blc. viridis* and characterize, at the single-molecule level, the mechanical unfolding process of the 4Hcyt subunit. Our study provides new insights into the strength of protein folding segments and intermolecular interactions of photosynthetic multiprotein complexes.

## 2 Materials and methods

### 2.1 Membrane preparation

*Blc. viridis* was grown anoxically in Medium 27 (DSMZ) at 30 °C under incandescent light as previously described [15], and harvested in late-log phase. Cells were harvested and rinsed with 2 mM Tris-HCl, 2 mM EDTA pH 7.5 and broken by French press followed by two centrifugation steps and a four-step (15%, 25%, 35%, 60%) sucrose gradient containing 0.03% n-Dodecyl β-D-maltoside (β-DDM), as is seen previously in the study of *Rba. sphaeroides* photosynthetic membranes [16]. The photosynthetic membranes were collected from the 60% green sucrose fraction.

### 2.2 Absorption spectra

Absorbance spectra were recorded on a UV-1600PC spectrophotometer (VWR) between 300 and 1100 nm.

### 2.3 SDS-PAGE

Samples of purified photosynthetic membranes were treated with SDS sample buffer (0.06 M Tris-HCl pH 6.8, 10% glycerol, 2% SDS, 0.1% bromophenol blue, 1.5% dithiothreitol) at 100 °C for 15–20 min. Proteins were loaded onto a 15% denaturing SDS-PAGE gel and run at 120 V for 45 mins. The gels were stained with Coomassie Blue stain for 20 mins and destained for 1 h.

### 2.4 Atomic force microscopy (AFM)

*Blc. viridis* photosynthetic membranes were immobilized on the mica substrate in 40 μl absorption buffer (10 mM Tris-HCl pH 7.2, 150 mM KCl, 25 mM MgCl_2_) for 1 h at room temperature. The sample was rinsed with imaging buffer (10 mM Tris-HCl pH 7.2, 150 mM KCl). AFM imaging was performed using a Bruker Multimode 8.0 equipped with a 97 μm J-scanner and OTR4-10 probe (spring constant = 0.08 N m^−1^) in PeakForce Quantitative Nanoscale Mechanical (PeakForce QNM) mode. Minimal loading forces of ~120 pN were used at scan frequencies of 3 Hz using optimized feedback parameters. High-speed AFM was carried out using a NanoWizard 3 AFM (JPK) equipped with an ULTRA S scanner and Ultra-Short Cantilever probe (0.3 N·m^−1^, Nano World) in AC mode with the scan frequency of 20–30 Hz. Image analysis was carried out using Gwyddion and ImageJ. Statistical data are presented as mean ± standard error of the mean (SEM) unless stated otherwise.

### 2.5 AFM-based single-molecule force spectroscopy (SMFS)

AFM has the great capacity of obtaining high-resolution topographic images and force measurements [17,18]. Force measurements were performed by repeating tip approach and retraction cycles with a z-ramp size of 200 nm, and at a tip velocity of 200 nm·s^−1^. The tip adhered non-specifically to the protein by a controlled loading force of ≤1 nN for ~1 s. As the tip is retracted from the membranes, the adhered protein is elongated until the external force overcomes the strength of the intra-/inter-molecular forces responsible for the integrity of the protein, resulting in unfolding of protein peptides. Many of the force curves had to be rejected because the force rupture events were incompatible with a polymer-unfolding model [19]. Finally, 278 curves (24% of all curves) were selected for subsequent analysis.

Data analysis of force measurements was carried out by SPIP™ software program (Image Metrology). The unfolding events in the force-distance curves were fitted using the worm-like chain (WLC) model, following:

$$F\left(x\right)=\frac{k_{B}T}{b}\left(\frac{1}{4{\left(1-\frac{x}{L}\right)}^{2}}-\frac{1}{4}+\frac{x}{L}\right)$$

where *F*(*x*) is the force at distance *x*, *k*_B_ is the Boltzmann constant, *b* is the average persistence length of polypeptides (assuming 0.4 nm), *L* is the contour length of the unfolded polypeptide chain, and *T* is the temperature = 298 K.

### 2.6 Structure representation and protein sequence analysis

The RC structure (PDB ID: 1PRC) [20] and RC-LH1 structure (PDB ID: 6ET5) [7] were analyzed using Pymol and PDBsum to carry out the structural representation. Protein sequences were acquired in Uniprot: 4Hcyt (Uniprot ID: P07173) and L subunits (Uniprot ID: P06009).

## 3 Results and discussion

### 3.1 Preparation of native photosynthetic membranes

**Fig. 2:**
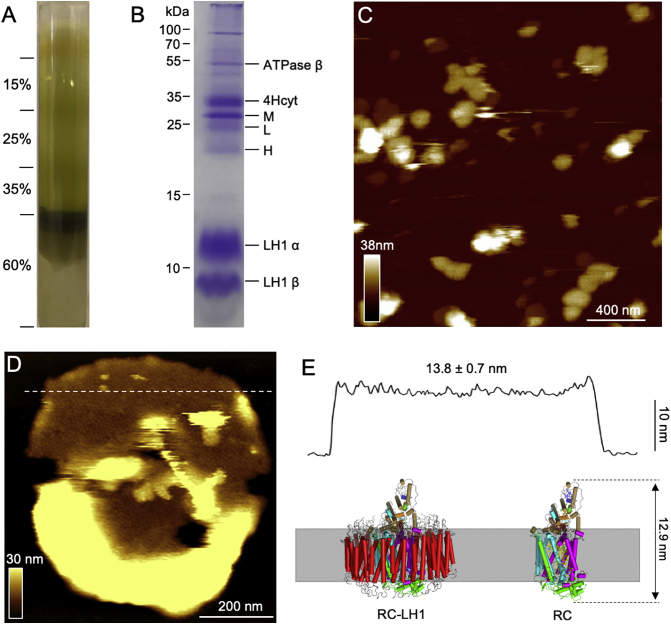
Isolation and AFM imaging of photosynthetic membranes from *Blc. viridis*. A. Step sucrose gradient centrifugation of the photosynthetic membranes, containing 0.03% n-Dodecyl β-D-maltoside (β-DDM). Photosynthetic membranes at the 35%–60% interface were extracted for further analysis. B. SDS-PAGE of the isolated photosynthetic membranes stained with Coomassie blue. The assigned components are labelled on the right. L: L subunit (PufL), M: M subunit (PufM), H: H subunit (PuhA), based on their molecular weights, consistent with the previous study [21]. C. AFM overview image of isolated *Blc. viridis* photosynthetic membrane patches. D. AFM topograph of an isolated photosynthetic membranes patch in liquid. E. Cross-section analysis along the dashed line in D reveals an average height of 13.8 ± 0.7 nm (*n* = 6) of the photosynthetic membranes containing the RC-LH1 complexes (bottom). The RC-LH1 and RC complexes were presented using the cryo-EM structure of RC-LH1 (PDB ID: 6ET5).

Photosynthetic membranes from *Blc. viridis* were isolated using sucrose gradient centrifugation (Fig. 2A), without freeze-thawing as reported previously [21]. The absorption spectrum of isolated photosynthetic membranes exhibits a characteristic absorption maximum at 1015 nm and a peak at 831 nm (Supplemental Fig. 1A), arising from BChl *b* in the RC–LH1 [7]. SDS-PAGE revealed the presence of the most predominate photosynthetic proteins based on their molecular weights (Fig. 2B), consistent with the previous study [21], demonstrating the structural integrity of the isolated photosynthetic membranes.

### 3.2 RC-LH1 complexes are densely packed in the photosynthetic membranes

The resulting photosynthetic membranes were then immobilized on freshly cleaved mica substrate for AFM imaging. AFM has demonstrated its extraordinary power in high-resolution imaging of native photosynthetic membranes [17,[22], [23], [24], [25]] and force measurements to delineate the mechanical unfolding of photosynthetic antenna membrane complexes [26]. AFM screening showed that the isolated photosynthetic membranes appear as two-dimensional patches with the sizes larger than 150 nm (Fig. 2C), allowing for AFM visualization of the long-range protein arrangement in the photosynthetic membranes. Fig. 2D shows a representative membrane patch that exhibits arrays of RC-LH1 complexes. Cross-section analysis (Fig. 2E) reveals that the membrane patches have an average thickness of 13.8 ± 0.7 nm (mean ± standard error of the mean (SEM), *n* = 6), comparable to the overall height of the RC-LH1 complex from the cytoplasmic face of the H subunit (Ser189) to the periplasmic extremity of the 4Hcyt subunit (Pro49) (12.9 nm, PDB ID: 6ET5) [7].

**Fig. 3:**
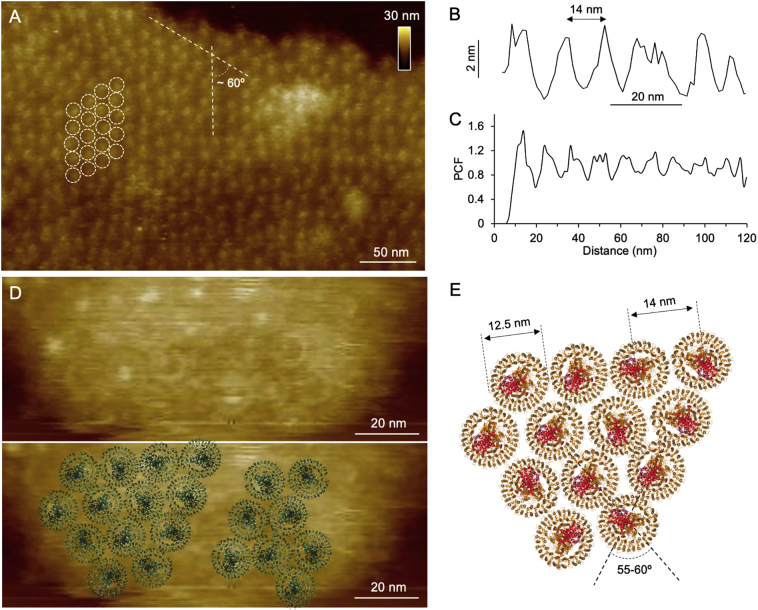
Arrangement of RC-LH1 complexes in photosynthetic membranes from *Blc. viridis*. A. Medium-resolution AFM image of the photosynthetic membrane in liquid. White circles indicate individual RC-LH1 complexes, revealing a regular array of core complexes, with an angle of ~60°. B. Height analysis indicates that the 4Hcyt heads are 14.8 ± 2.6 nm apart in the periplasmic surface of photosynthetic membranes. C. Pair correlation function (PCF) analysis of the RC-LH1 distribution in the periplasmic surface reveals a ~14 nm distance between adjacent RC-LH1 complexes. D. High-resolution image of the photosynthetic membranes in liquid demonstrates a compact arrangement of RC-LH1 complexes (upper). The cryo-EM structure of RC-LH1 (PDB ID: 6ET5) is superimposed in the AFM image (lower). E. Schematic arrangement of RC-LH1 complexes as revealed by AFM imaging. Each RC-LH1 complex is 12.5 nm in size and the space between neighbouring RC-LH1 complexes is ~14 nm.

Closer AFM inspection provided an overview of the distribution of RC-LH1 complexes in the photosynthetic membranes (Fig. 3A). On the periplasmic surface, the RC-LH1 complexes, represented as individual protruding particles due to the presence of 4Hcyt, are densely packed and form regular arrays in a ~60° staggered pattern, resulting from the inherent circular structures packing in the densest manner. The protruding height of 4Hcyt above the membrane surface is 5.1 ± 0.7 nm (*n* = 32) (Fig. 3B), similar to the protrusion of 4Hcyt above the membrane plane as indicated by the reported cryo-EM structure [7]. The distance between adjacent RC-LH1 complexes is 14.8 ± 2.6 nm (*n* = 30) (Fig. 3B), consistent with the pair correlation function results that indicate the closest distance between two RC-LH1 complexes of ~14 nm (Fig. 3C). Higher-magnification AFM topograph illustrates the structures of individual circular RC-LH1 complexes and their lateral arrangement in the membrane, with the angle between RC-LH1 arrays of 55-60^o^ (Fig. 3D, E). During high-resolution AFM imaging, the 4Hcyt heads could be removed by scanning forces exerted by the AFM tip, resulting in better characterization of the associated LH1 organization (Fig. 3D). The density of RC-LH1 complexes and the space between neighbouring RC-LH1 complexes differ in different membrane regions, implying the inherent variability of the protein organization of these photosynthetic membranes (Supplemental Fig. 2).

RC-LH1 complexes account for over 90% of the total proteins in the *Blc. viridis* photosynthetic membranes [27]. In such a highly crowded membrane environment, RC-LH1 complexes are presumed to possess restricted diffusion due to the lack of space in the membrane. High-speed AFM imaging confirmed the highly stable organization of RC-LH1 complexes. No detectable lateral movement of the strongly protruded 4Hcyt heads was observed during continuous AFM scanning over 85 s (Supplemental Fig. 3), indicating a higher restriction of protein diffusion than those of packed membrane proteins and self-assembling proteins [[28], [29], [30]], although the influences caused by sample-substrate absorption cannot be discounted.

### 3.3 Mechanical unfolding of the 4Hcyt subunit

**Fig. 4:**
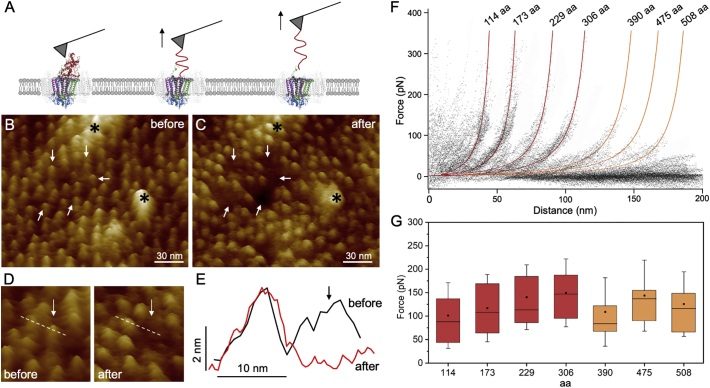
Force measurements of 4Hcyt unfolding. A. Schematic representation of single-molecule force spectroscopy (SMFS) on the *Blc. viridis* RC-LH1 complex. B and C. AFM images of the photosynthetic membrane fragment before and after force measurement. Medium-resolution AFM images allow precise alignment of images (stars indicate unchanged protein complexes). Force measurements have removed and unfolded the 4Hcyt subunits from the RC-LH1 complexes (white arrows). D. An example of the mechanical removal of a protruding 4Hcyt head. E. Height analysis along the dashed lines in D confirms the removal of the 4Hcyt subunit. F. Superimposed force-distance curves, each recorded upon mechanically unfolding a single 4Hcyt subunit from the RC-LH1 complex in the native membrane, reveal the unfolding pattern of 4Hcyt. Red and orange force-extension curves are Worm-Like Chain (WLC) curves, indicating the mean contour lengths of seven detected force peaks in the force-distance curves, 114, 173, 229, 306, 390, 475, and 508 amino acids (aa). G. Forces required in individual unfolding steps are 101.2 ± 9.7 pN for 114 aa (*n* = 62), 117.1 ± 12.1 pN for 173 aa (*n* = 42), 140.3 ± 12.4 pN for 229 aa (*n* = 37), 149.5 ± 18.1 pN for 306 aa (*n* = 19), 108.9 ± 18.9 pN for 390 aa (*n* = 18), 143.7 ± 21.0 pN for 475 aa (*n* = 15), and 125.7 ± 28.1 pN for 508 aa (*n* = 7) (see Table 1).

By applying the scanning force of ~200 picoNewtons (pN) during AFM imaging, previous studies have reported that the globular 4Hcyt structure could be mechanically removed [21], indicating its flexible binding with the RC subunits. To examine the mechanical properties of 4Hcyt, AFM-based single-molecule force measurement was exploited to unfold the 4Hcyt heads of the RC-LH1 complex in the native membrane environment. The C-terminal end of 4Hcyt is exposed away from periplasmic side of the photosynthetic membranes (Supplemental Fig. 4). We placed the AFM probe onto a *Blc. viridis* photosynthetic membrane patch to non-specifically attach the C-terminus of 4Hcyt with a controlled loading force ≤1 nN for ~1 s. Then the probe was retracted from the membrane surface at a constant velocity of 200 nm·s^−1^ (Fig. 4A) and the force was recorded as a function of the distance between the AFM probe and membrane surface (shown as force-distance curves).

AFM topographic analysis of the membranes before and after force measurements allowed us to determine the specific unfolding events. Individual RC-LH1 complexes were visualized in their associations with neighbouring RC-LH1 structures prior to force measurements (Fig. 4B). Imaging the same membrane region after force measurements and cross-section analysis demonstrated the physical removal of 4Hcyt subunits from the RC-LH1 complex in the native photosynthetic membrane (Fig. 4C-E).

The force-distance curves that were compatible with a polymer-unfolding model [19] were finally selected for further analysis (Supplemental Fig. 5). Superposition of 200 force curves reveals a pattern of seven pronounced peaks, corresponding to 7 unfolding events (Fig. 4F), revealing a consistent unfolding pattern. By fitting each of the 7 force peaks with the Worm-Like Chain (WLC) model using a persistence length of 0.4 nm (the approximate length of amino acid residues), we determined the contour lengths of the polypeptide segments unfolded in each unfolding event: 114, 173, 229, 306, 390, 475, and 508 amino acids (Fig. 4F, Table 1).

**Table 1:** Analysis of the unfolding events.

Contour length	Force (pN)	Unfolding subunit	Residues	Helices
114 aa	101.2 ± 9.7	4Hcyt	Gly349C-Arg236C	H1-H8
173 aa	117.1 ± 12.1	4Hcyt	Ser235C-Arg177C	H9-H10
229 aa	140.3 ± 12.4	4Hcyt	Asn176C-Lys121C	H11-H12
306 aa	149.5 ± 18.1	4Hcyt	Ala120C-His44C	H13-H16
390 aa	108.9 ± 18.9	4Hcyt, L	Leu43C-Cys21C Asp269L-Thr209L	H1-H3
475 aa	143.7 ± 21.0	L	Lys208L-Cys123L	H4-H5
508 aa	125.7 ± 28.1	L	Phe122L-Ile90L	H6

The forces required in individual unfolding steps within the 4Hcyt subunit are 101.2 ± 9.7 pN for 114 aa (*n* = 62), 117.1 ± 12.1 pN for 173 aa (*n* = 42), 140.3 ± 12.4 pN for 229 aa (*n* = 37), and 149.5 ± 18.1 pN for 306 aa (*n* = 19) (Fig. 4G, Table 1). An average rupture force is approximately 121.2 ± 5.6 pN (*n* = 200), with comparable magnitudes as those determined for unfolding of the muscle protein titin [31], the extracellular matrix glycoprotein tenascin [32], the transmembrane protein in an LH2 complex [26] and bacteriorhodopsin [33] at similar pulling velocities. The determined rupture force is slightly lower than the AFM scanning force of 200 pN, explaining to some extent why AFM nanodissection could cause removal of 4Hcyt [21]. In addition, the rupture force increases as more of the peptide fragments are unfolded (Fig. 4G), indicative of an increase in the strength of each consecutive structural segment. The locations of these structural components are specified below.

**Fig. 5:**
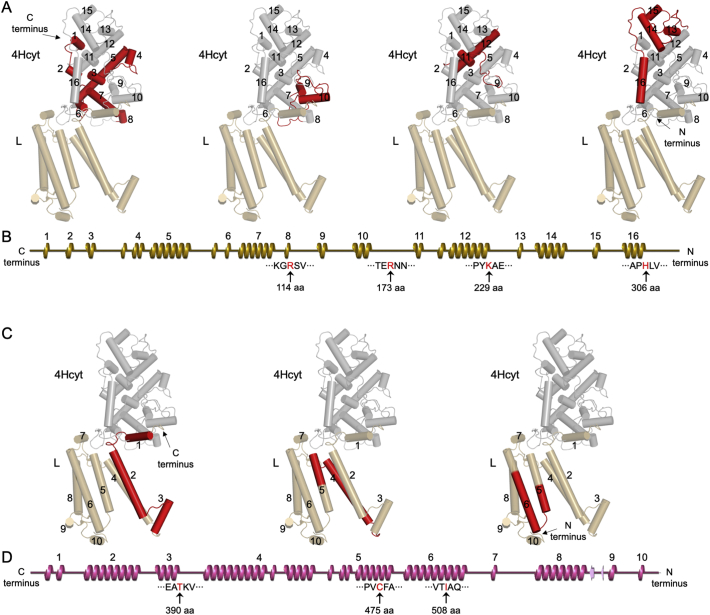
Proposed unfolding pathways of the *Blc. viridis* RC-LH1 4Hcyt and L subunits. A. Sequential unfolding of the 4Hcyt subunit (grey) of the RC complex (PDB ID: 1PRC), starting from the C-terminal end to the N-terminus. The 15 α-helices of 4Hcyt are numbered and the structural segments unfolding in each unfolding step are highlighted in red. The interacting L subunit is shown in brown. B. Secondary structure model of the 4Hcyt subunit with the residues (red, indicated by arrows) unfolded in each unfolding step. The 15 α-helices of 4Hcyt are numbered. C. Sequential unfolding of the L subunit (brown) of the RC complex induced by 4Hcyt unfolding, starting from the C-terminal end to the N-terminus. The N-terminal end of the 4Hcyt subunit interacts with the first α-helix at the C-terminus of the L subunit. The structural segments unfolding in each unfolding step are highlighted in red. D. Secondary structure model of the L subunit with the residues (red, indicated by arrows) unfolded in each unfolding step. The 10 α-helices of the L subunit are labelled.

To investigate the detailed gradual unfolding pattern, we analyzed the secondary and three-dimensional structures of the *Blc. viridis* 4Hcyt subunit (Fig. 5, Supplemental Fig. 6). The 4Hcyt subunit is 356 amino acids long (Uniprot ID: P07173). The predominant elements of the 4Hcyt secondary structure are α-helices, and β-sheets are rare. Residues Lys356C to Pro350C (C represents the PufC subunit) at the C-terminus of the Cyt subunit are completely disordered and were not structurally solved [20]. The tip-sample separation led to unfolding of the 4Hcyt subunit from the C-terminus. The first peak of the force curve displays an unfolding length of 114 aa (Fig. 4F), revealing the unfolding of α-helices 1–8 of the 4Hcyt subunit from residues Gly349C to Arg236C (Fig. 5A, B, Supplemental Fig. 6, Table 1). This unfolding event requires approximately 101.2 pN. Subsequently, the α-helices 9–10 (Ser235C-Arg177C), 11–12 (Asn176C-Lys121C) and 13–16 (Ala120C-His44C) were unfolded, corresponding to the second, third, and forth peaks of the force curve with the unfolding length of 173, 229, and 306 aa, respectively (Fig. 5A, B, Supplemental Fig. 6, Table 1). The covalent binding of heme to the 4Hcyt protein subunit might perturb these contour lengths slightly.

### 3.4 Unfolding of 4Hcyt could result in unfolding of the RC L subunit

Interestingly, there are only 328 amino acids from the first α-helix at the C-terminus (Gly349C) to the Cys21C at the N-terminal region of the 4Hcyt subunit. The occurrence of the remaining three unfolding events with contour lengths of 388, 475 and 508 aa (fitted with orange curves) indicated that the unfolding of 4Hcyt resulted in the unfolding of the transmembrane RC L and M subunits that interact with 4Hcyt. Structural analysis revealed that the Cyt residues in the connecting sequences between heme-binding segments form interactions with the M subunit, whereas the Cyt N-terminal segment (residues 1–40) contacts only with the L subunit [20]. In particular, the residues Cys21C and Glu23C have close contacts with the first α-helix from the C-terminus of the L subunit (Supplemental Fig. 4).

The fifth force peak with the unfolding length of 390 aa indicates that pulling the 4Hcyt peptide by the AFM probe resulted in unfolding of α-helices 1–3 from residues Asp269L to Thr209L (L represents the PufL subunit) (Fig. 5C, D). This unfolding event requires a force of 108.9 ± 18.9 pN (*n* = 18). The last two unfolding events occur with the unfolding lengths of 475 and 508 aa, corresponding to the unfolding of α-helices 4–5 (Lys208L-Cys123L) and the α-helix 6 (Phe122L-Ile90L) with the required forces of 143.7 ± 21.0 pN (*n* = 15) and 125.7 ± 28.1 pN (*n* = 7), respectively. These results demonstrate that the interacting force between the 4Hcyt N-terminus and the C-terminus of the L subunit is larger than the maximum unfolding force detected in unfolding of the L subunit (143.7 pN). The strong binding between 4Hcyt and the transmembrane RC subunits may provide the foundation for efficient electron flow. It is also worth noting that the chance to record longer unfolding events over 306 aa is lower than shorter unfolding events, suggesting that the L subunit could break at any amino acid residue during the unfolding events due to the unspecific binding between the AFM probe and 4Hcyt and relatively weak interactions between 4Hcyt and the L subunit. This may explain the fact that only partial unfolding of α-helices 5 and 6 was detected in the 475 and 508 aa unfolding events (Fig. 5C, D).

In some purple photosynthetic bacteria that lack the 4Hcyt subunit in the RC complex, the soluble electron carrier Cyt *c*_2_ docks directly to the RC complex through electrostatic interactions [34]. The specific binding has allowed the recognition of the *Rba. sphaeroides* RC complexes using the AFM probes pre-modified with Cyt *c*_2_ [35]. Interestingly, the measured unbinding force was above 160 pN, higher than the average force required for the unfolding of the 4Hcyt subunit. It remains unclear whether the AFM-induced unbinding of Cyt *c*_2_ from the RC could result in unfolding of RC subunits.

The CysC-1 at the N-terminus of the *Blc*. viridis 4Hcyt subunit (Fig. 5A) has been characterized to bind with a covalently bound lipid through a thioether bond and function as a membrane anchor for 4Hcyt to associate with the periplasmic surface of the photosynthetic membrane prior to interacting with other RC subunits [5,36,37]. However, the binding between 4Hcyt and the lipid membrane may be relatively small to measure in SMFS.

## 4 Conclusions

Using AFM imaging and single-molecule force spectroscopy, this study provides quantitative insight into the organization of photosynthetic complexes and the assembly mechanisms of the RC complex from the BChl *b*-producing purple bacterium *Blc. viridis*. The RC-LH1 complexes are densely organized in native photosynthetic membranes. Mechanical unfolding of the globular 4Hcyt subunit at the periplasmic side of the RC represents a stepwise process. Removal of the 4Hcyt subunit also resulted in the unfolding of the L subunit of the RC complex, indicating strong binding of the non-membranous 4Hcyt structure to the RC transmembrane subunit. We anticipate that our approach will provide a means of studying the folding and interactions of membrane-associated proteins and the assembly of membrane protein complexes at the single-molecule level, allowing the exploration and reprogramming of native and synthetic biological membranes.

## Author contributions

L.C.M. and L.-N.L. designed the experiments; L.C.M. and L.Z. performed experiments; L.C.M., D.M. and L.-N.L. analyzed data; L.C.M., D.P.C., D.M. and L.-N.L. wrote the manuscript.

## Declaration of competing interest

The authors declare that they have no known competing financial interests or personal relationships that could have appeared to influence the work reported in this paper.

## Appendix A Supplementary data

Supplementary figures Image 1
