# Supplementary material for: Unfolding pathway and intermolecular interactions of the cytochrome subunit in the bacterial photosynthetic reaction center
Source: Biochim Biophys Acta Bioenerg. 2020 Aug 1;1861(8):148204. doi: 10.1016/j.bbabio.2020.148204 (PMC7322399; doi:10.1016/j.bbabio.2020.148204)
Supplement: Supplementary file 1 — Supplementary figures [file mmc1.docx]

**Supplemental Information**

**for**

**Unfolding pathway and intermolecular interactions of the cytochrome subunit in the bacterial photosynthetic reaction center**

Leanne C. Miller^1,2^, Longsheng Zhao^1,3,4^, Daniel P. Canniffe^1^, David Martin^2^, Lu-Ning Liu^1,5,^*

^1^ Institute of Integrative Biology, University of Liverpool, Liverpool L69 7ZB, United Kingdom

^2^ Department of Physics, University of Liverpool, Liverpool L69 7ZE, United Kingdom

^3^ State Key Laboratory of Microbial Technology, Marine Biotechnology Research Center, Shandong University, Qingdao 266237, China

^4^ Laboratory for Marine Biology and Biotechnology, Pilot National Laboratory for Marine Science and Technology, Qingdao 266237, China

^5^ College of Marine Life Sciences, and Frontiers Science Center for Deep Ocean Multispheres and Earth System, Ocean University of China, Qingdao 266003, China

*Corresponding Author: Lu-Ning Liu; address: Institute of Integrative Biology, University of Liverpool, Liverpool L69 7ZB, United Kingdom; Email: [Luning.Liu@liverpool.ac.uk](mailto:Luning.Liu@liverpool.ac.uk)

**
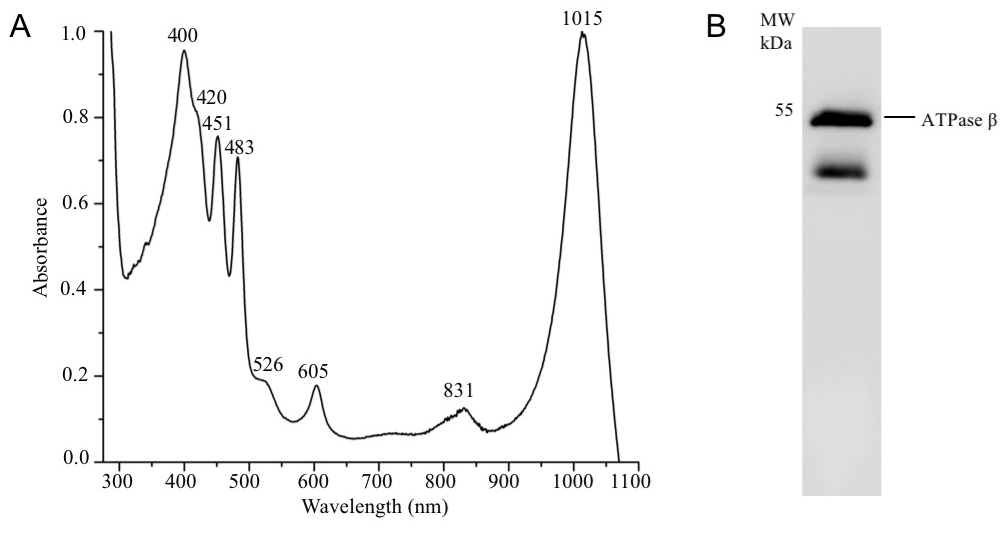
Supplemental Fig. 1. Room-temperature absorption spectrum of isolated photosynthetic membranes from *Blc. viridis.***


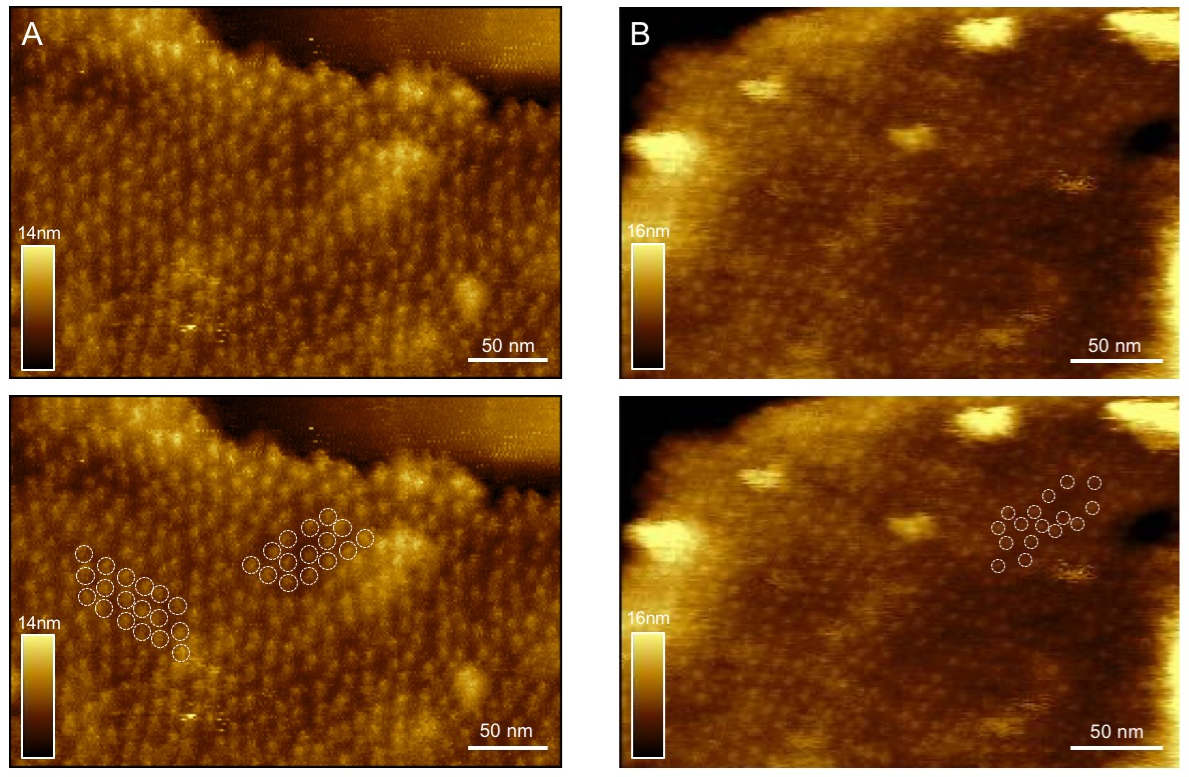


**Supplemental Fig. 2. Variability of the distribution of RC-LH1 complexes in native photosynthetic membranes. A**. A densely-packed photosynthetic membrane patch containing 112 RC-LH1 complexes per 15640 nm^2^, covering ~81% of the total membrane. The RC-LH1 complexes are 14.8 ± 2.6 nm apart. Circles indicate individual 4Hcyt heads. **B**. A less densely-packed photosynthetic membrane patch containing 73 RC-LH1 complexes per 15640 nm^2^, covering 53% of the total membrane. The RC-LH1 complexes are 17.9 ± 5.6 nm (*n* = 70) apart. Circles indicate individual 4Hcyt heads.


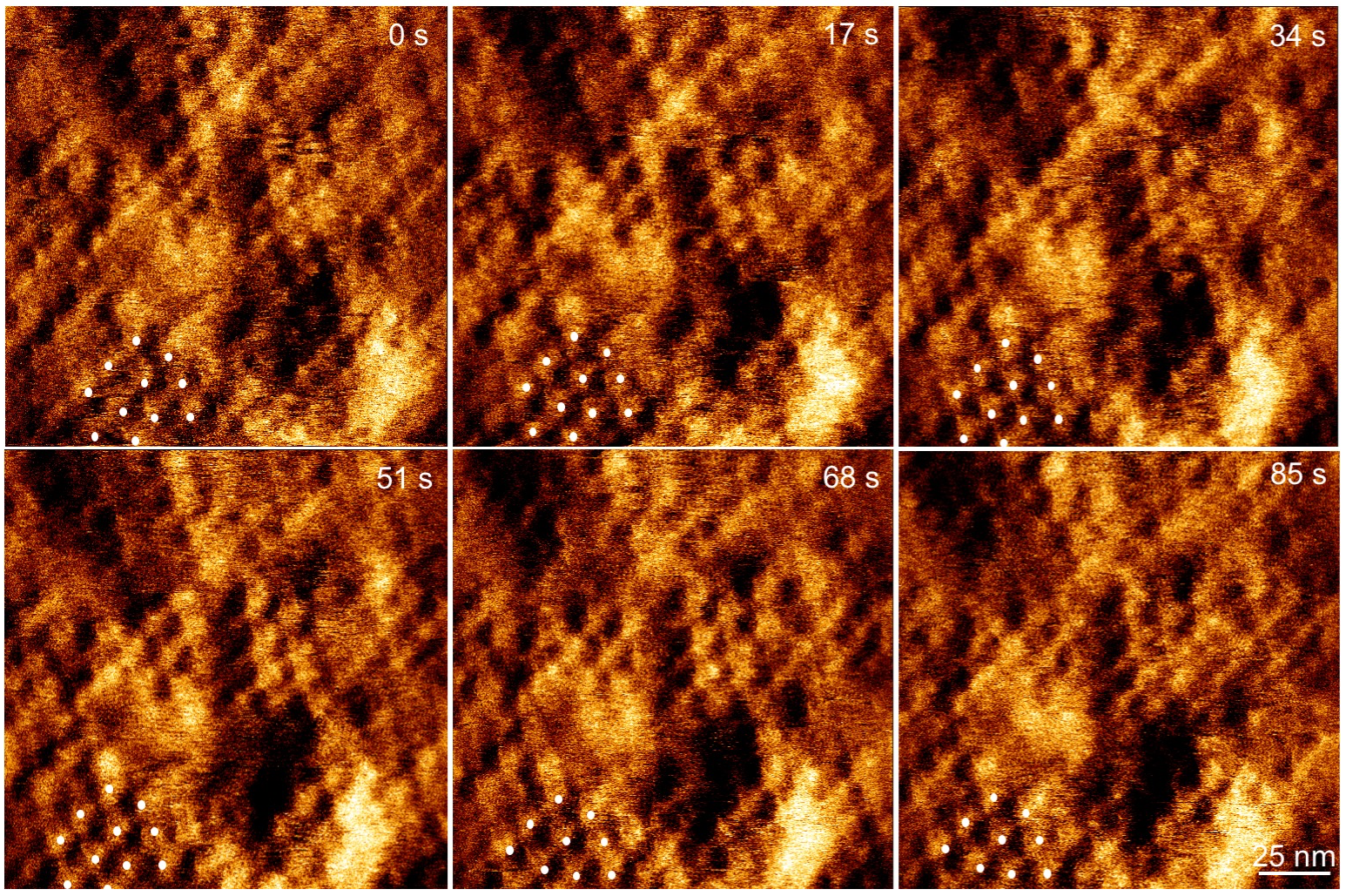


**Supplemental Fig. 3. High-speed AFM imaging reveals the stable organization of RC-LH1 complexes in isolated photosynthetic membranes.** Time-lapse AFM amplitude error signal images were captured at 17 sec per frame, revealing no detectable changes in the 4Hcyt subunit (white dots) organization within 85 sec.


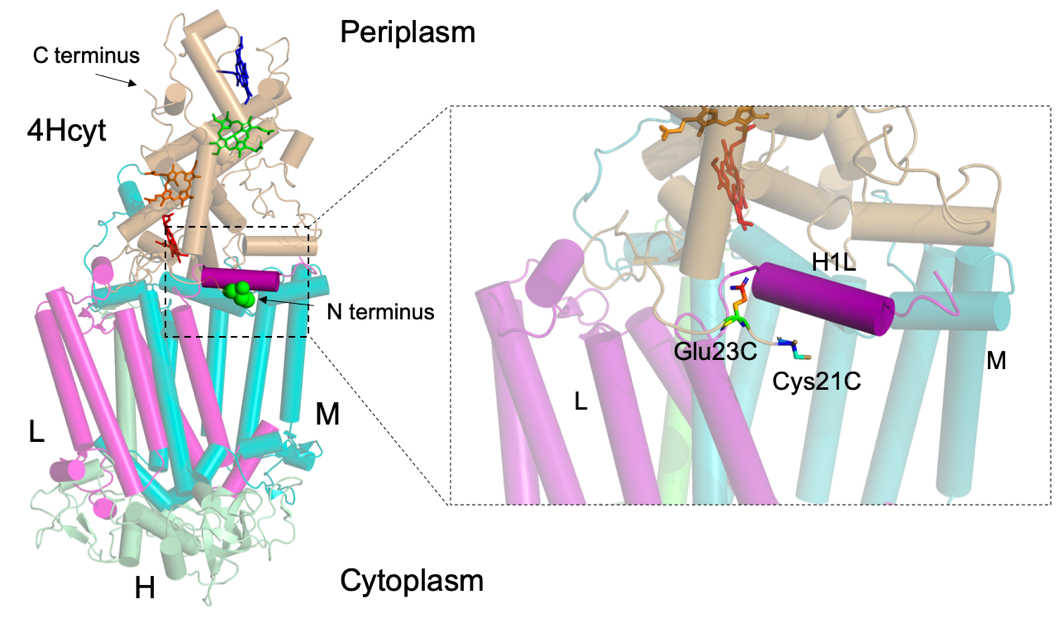


**Supplemental Fig. 4. Interactions of 4Hcyt with the L subunit in the RC complex.** The structure was presented using the atomic structure of RC (PDB ID: 1PRC). The four colored heme groups are shown in sticks. The residues Cys21C and Glu23C at the N-terminal end of 4Hcyt have close contacts with the first α-helix from the C-terminus of the L subunit.


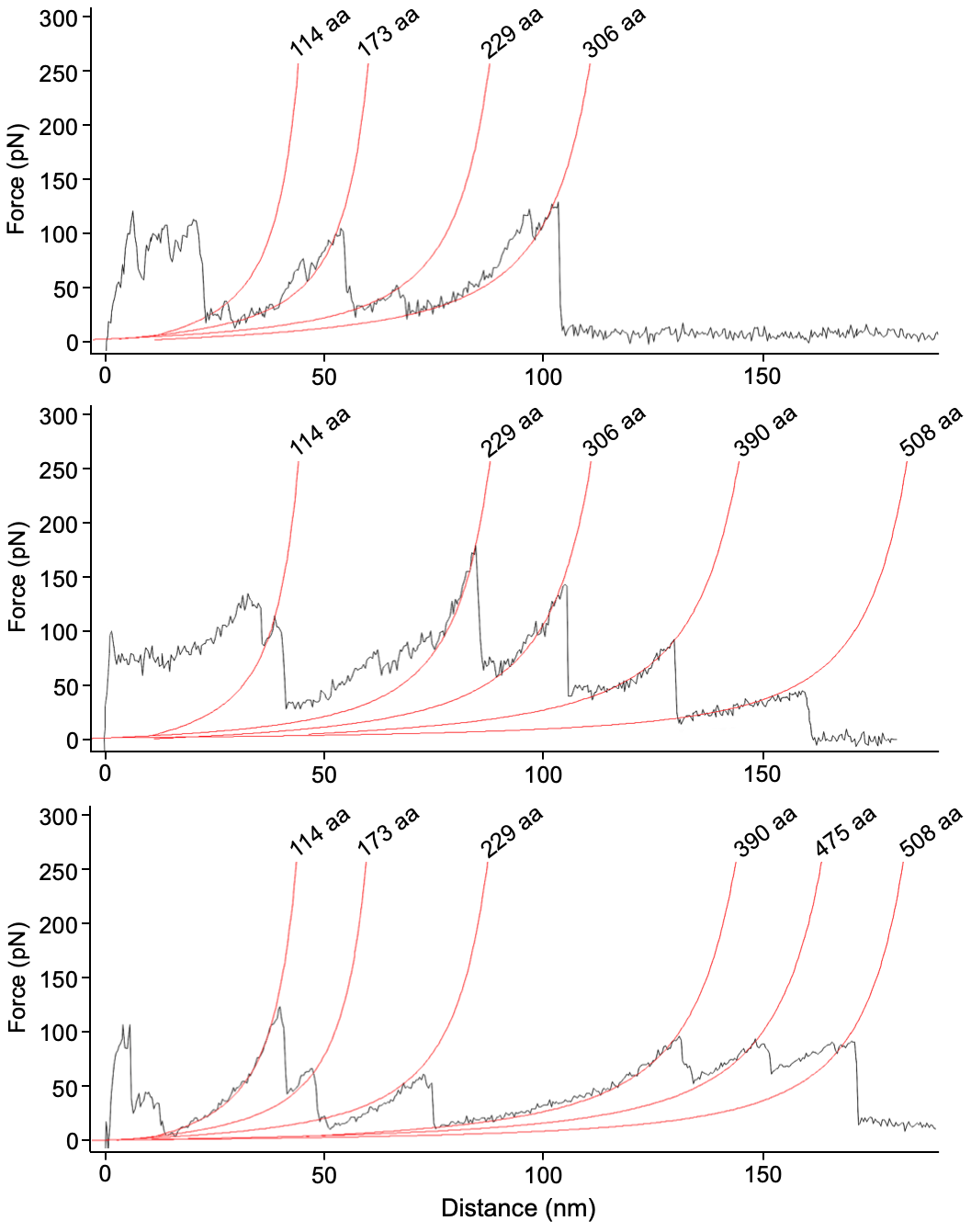


**Supplemental Fig. 5. Selected single force-distance curves (black) with individual peaks fitted by the WLC model (red) denote different structural intermediates in the unfolding pathways.** The mean contour lengths of detected force peaks are indicated.


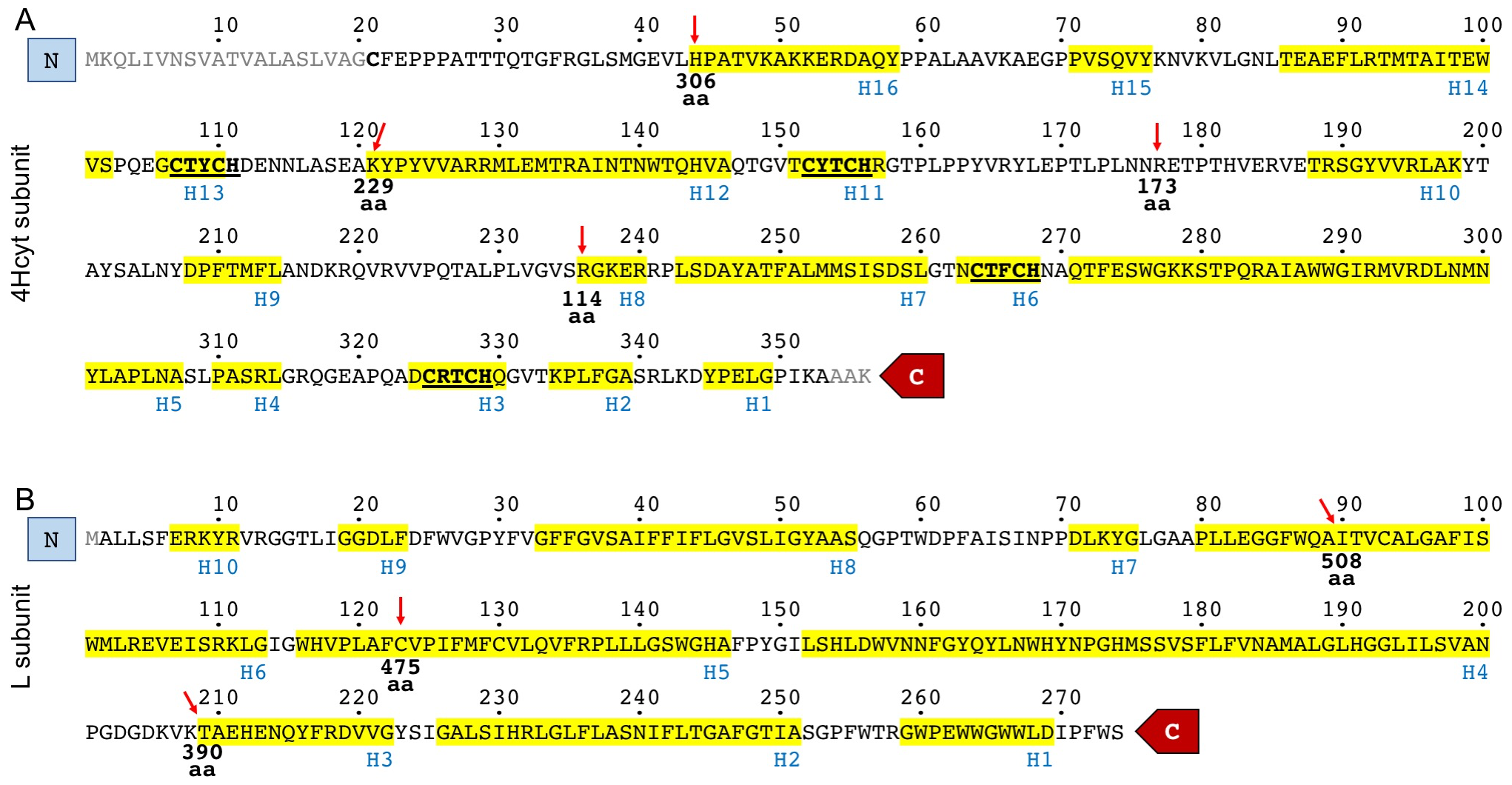


**Supplemental Fig. 6. Peptide sequence analysis of the *Blc. viridis* 4Hcyt and L subunit.** Protein sequences were acquired in Uniprot: 4Hcyt (Uniprot ID: P07173) and L subunits (Uniprot ID: P06009). The α-helices are highlighted in yellow and numbered. Red arrows indicate the key residues in the unfolding events. The heme-binding residues were indicated by underlines.
